# Innovations in Practice: Beyond the clinic – embracing natural environments in mental health care for children and young people

**DOI:** 10.1111/camh.12768

**Published:** 2025-06-04

**Authors:** David Francis Hunt, Gemma R. Morshead, Rachel Hayes, Siobhan Mitchell, Beth Chapman, Carl Dutton, Anna Adlam, Catriona Mellor

**Affiliations:** ^1^ Department of Psychology University of Exeter Exeter UK; ^2^ Cornwall Partnership NHS Foundation Trust Cornwall UK; ^3^ NIHR Applied Research Collaboration South West Peninsula (PenARC), University of Exeter Exeter UK; ^4^ Alder Hey Childrens NHS Foundation Trust Liverpool UK; ^5^ Natural Academy Bristol UK; ^6^ Oxford Health NHS Foundation Trust Oxford UK

**Keywords:** Environment, evaluation, mental health, qualitative methods, child care

## Abstract

**Background:**

Engaging children and young people (CYP) with natural environments while in healthcare can offer significant therapeutic benefits. Despite evidence supporting nature‐based interventions, their use in CYP healthcare settings remains inconsistent. This article outlines how to integrate an eco‐biopsychosocial model in healthcare, combining ecological aspects with traditional biopsychosocial frameworks to improve health outcomes, placing CYP within their social and ecological contexts, and promoting holistic, person‐centred care.

**Methods:**

Through a series of meetings, clinicians and practitioners involved in nature‐based approaches (NBAs) projects in the UK shared their expertise. They reflected on their experiences and identified patterns in the learning related to implementing these activities and embedding them into practice. The projects included: (1) Oxford Health NHS Foundation Trust. (2) Cornwall Partnership NHS Foundation Trust. (3) Alder Hey Children's NHS Foundation Trust.

**Results:**

We discuss the importance of NBAs within national healthcare frameworks and provide successful examples from Oxford Health NHS Foundation Trust, Cornwall Partnership NHS Foundation Trust and Alder Hey Children's NHS Foundation Trust. These case studies highlight the potential for a positive impact on wellbeing, resilience and staff satisfaction. Key strategies for implementing the model include relational, procedural, and environmental factors for creating a culture of nature‐based practice.

**Conclusions:**

This innovation in practice article emphasises the need for governance, evaluation and dissemination to ensure the sustainability of these initiatives. By adopting this integrative approach, we aim to reduce health inequalities and promote a shift towards a nature‐inclusive strategy in healthcare, which promotes the long‐term wellbeing of CYP as well as supports more sustainable services.

**Key Practitioner Messages**

**What is known?**

- There are several benefits to incorporating nature‐based approaches in CYP healthcare settings for patient experience and outcomes.
- The implementation of nature‐based approaches in practice is not well known.

**What is new?**

- This article provides knowledge and considerations for integrating nature‐based approaches into standard practice, drawing insights from three projects across the UK.

**The direct relevance of the reported work to clinical practice in child and adolescent mental health**

- This article aims to explore future pathways for nature‐based approaches in research and to serve as a guide for service improvements in CYP healthcare settings.

## Introduction

Emerging evidence shows that engaging children and young people (CYP) with natural environments – typically defined as either a physical setting primarily shaped by natural processes (e.g. an ecosystem) or a humanmade space with natural elements (e.g. a garden) – offers significant mental, physical and social benefits (Arola et al., 2023; Mellor, Botchway, Barnes, & Gandy, 2022; Mygind et al., 2019). National healthcare bodies recognise the importance of nature in their services (NHS England, n.d.; Royal College of Psychiatrists, 2024), as interactions with nature facilitate a shift towards de‐medicalised, person‐centred, holistic therapeutic approaches (Wheeler, Gordon‐Brown, & Lovell, 2020; Shanahan et al., 2019).

Despite this, integrating nature‐based approaches (NBAs) – defined as using natural environments and/or elements, such as outdoor activities or nature interactions, to support mental health and wellbeing – into healthcare services is inconsistent, demonstrating the need for a structured approach via the eco‐biopsychosocial model (Lovell, 2016). Examples of NBAs include formal activities such as forest schools and informal activities such as engaging in care planning through ‘walk and talk’ sessions. This model integrates ecological elements with the traditional biopsychosocial framework, emphasising the role of natural environments in healthy development (White et al., 2023), providing a comprehensive framework for understanding and addressing healthcare by situating individuals within social and ecological contexts.

With the shift in the needs of CYP against the backdrop of climate emergencies and overstretched healthcare systems, it is important to recognise the need to support them in ways that support their specific challenges. This is illustrated by the i‐Thrive model, which is a person‐centred, needs‐led approach to mental health care for CYP, focusing on prevention, shared decision‐making and providing support across five categories: thriving, advice, help, more help and risk support (Wolpert et al., 2019).

As healthcare systems adapt to shifting needs, NBAs offer a way to support CYP in a holistic and responsive manner. Examining how these approaches have been implemented in different settings can provide insight into their potential and the challenges involved.

## The need for nature‐based approaches (NBAs) in CYP healthcare

Burgeoning evidence supports integrating NBAs in healthcare for their positive impact on CYP wellbeing (Bratman et al., 2019; Richardson, Richardson, Hallam, & Ferguson, 2020). Although mechanisms are unclear (Rowley, Topciu, & Owens, 2022), these approaches reduce stress (Yao, Zhang, & Gong, 2021), enhance wellbeing and improve physical health through engagement with nature and opportunities for enhancing therapeutic relationships in less institutionalised settings (Capaldi, Dopko, & Zelenski, 2014; Hunt, Morgan, Connors, & Mellor, 2022).

NBAs also support systemic approaches, enhancing familial bonding, providing opportunities for CYP to connect with their families and exploring new activities which can strengthen relationships and improve communication (Tillman, Tobin, Avison, Tillmann, & Gilliland, 2018; Vanaken & Danckaerts, 2018). Promoting diverse NBAs can bridge the gap in access to natural spaces, benefiting CYP from urban environments and low socioeconomic backgrounds who may have little access to green spaces. Addressing inequitable access is vital, as the COVID‐19 pandemic highlighted these disparities, with disadvantaged households spending less time outdoors (Natural England, 2021). Ensuring equitable access to NBAs requires strategic resource allocation, considering underserved groups within an eco‐biopsychosocial framework. Immersive technologies present opportunities to explore the impact of nature on CYP health and wellbeing virtually (Owens & Bunce, 2023).

In addition to these advantages, NBAs are particularly relevant in addressing the climate and ecological crises. Eco‐distress, characterised by emotional responses to these crises, is an emerging concern, and it is therefore important that healthcare adapts to integrate nature into practice (Crandon, Scott, Charlson, & Thomas, 2022). Research suggests that developing self‐efficacy and agency can help mitigate these challenges (Innocenti et al., 2023). As such, incorporating natural environments into healthcare through an eco‐biopsychosocial approach can play a dual role in meeting the changing landscape of patient needs while promoting long‐term resilience and environmental stewardship (Hickman et al., 2021).

This article – guided by the eco‐biopsychosocial model – aims to provide practical guidance on implementing NBA projects. Drawing on our expertise as researchers and clinicians who have led these projects across three locations in England, reflecting on and synthesising the key lessons learned from our work.

## Methods

The practical guidance presented in this article was developed through a series of discussions within a community of practice across three projects focused on delivering NBA approaches in CYP settings. Authors, alongside the broader community of practice, reflected on the progress and outcomes of each project, synthesising the learning into the identified themes and positioning these within the conceptual framework of the eco‐biopsychosocial approach to healthcare (as described below).

### Conceptual framework

**Figure 1:**
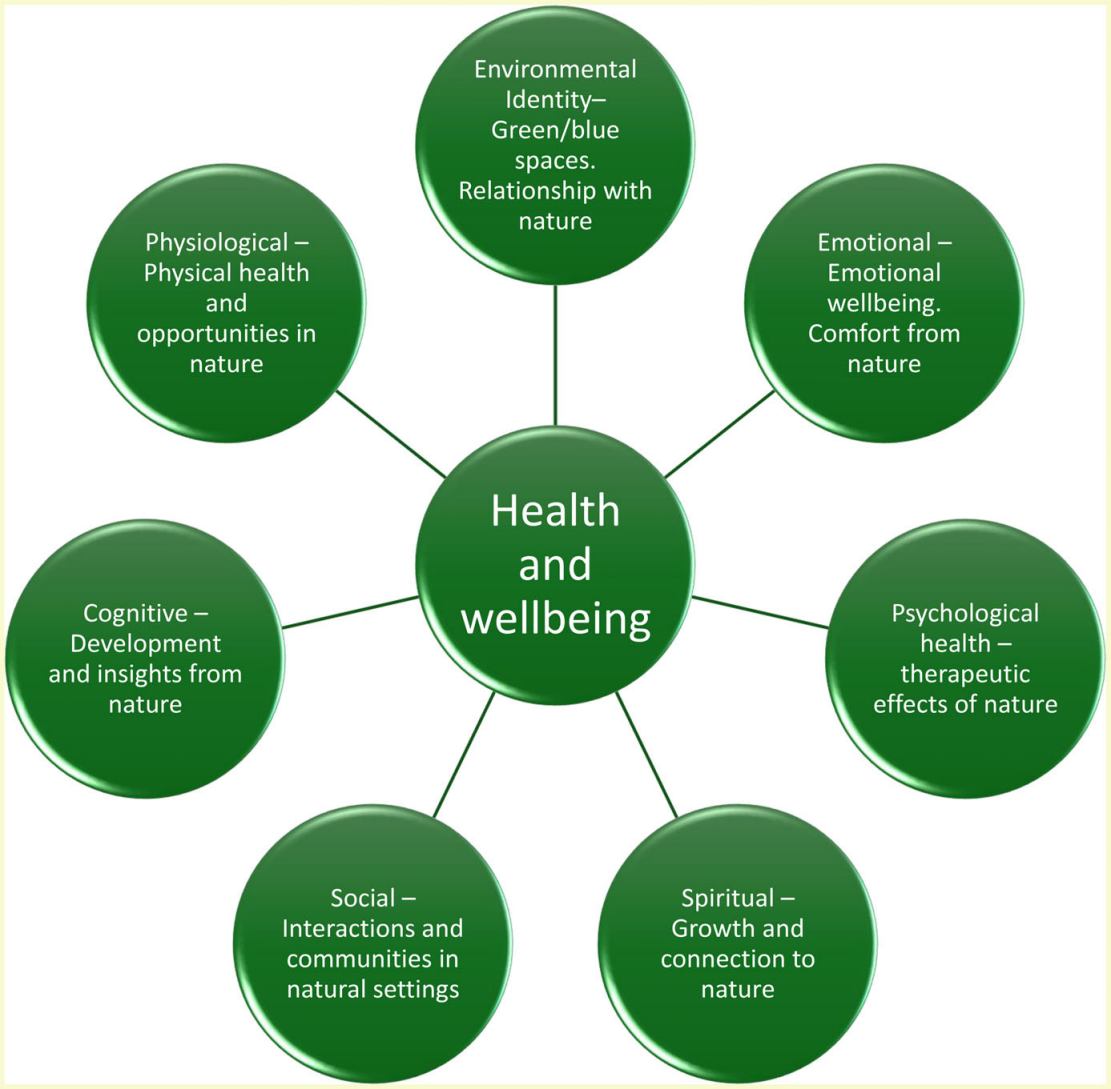
Overview of the eco‐biopsychosocial model. The outer circles relate to the different facets of health and wellbeing from the eco‐biopsychosocial approach

An eco‐biopsychosocial perspective recognises the fundamental role of nature in human development across various domains (Arola et al., 2023; Lumber, Richardson, & Sheffield, 2017; White et al., 2023). Rooted in the One Health approach, it highlights the interconnected health of humans, animals and the environment (Pitt & Gunn, 2024). Figure 1 illustrates humans as integral parts of nature, highlighting opportunities for wellbeing. This perspective demonstrates the benefits of having access to green and blue spaces and the importance of nature connection, encompassing wellbeing, meaning, purpose, joy, comfort and insight. It includes both external (e.g. animals, landscapes) and internal (e.g. bodies, emotions) elements.

In practice, an eco‐biopsychosocial perspective can be applied in assessments, management and self‐care, addressing health inequalities by recognising unequal access to nature. Nature can serve as a co‐therapist, transforming the outdoors into an accessible therapy space for long‐term wellbeing. This approach also identifies nature‐based practitioners as allies in supporting CYP, nurturing a holistic approach that enhances individual health and deepens connection to the natural world.

## Case studies

Next, we present three projects from Oxford Health NHS Foundation Trust, Cornwall Partnership NHS Foundation Trust and Alder Hey Children's NHS Foundation Trust that embody an eco‐biopsychosocial approach, conducted by different teams that formed a core working group to discuss their approaches.

### Swindon CAMHS ‘Greener Marlborough House’ (Oxford Health NHS Foundation Trust) project: Incorporation of nature in inpatient settings

The ‘Healthy Lives’ initiative at Marlborough House – a Child and Adolescent Mental Health (CAMHS) service – prioritises foundational elements of wellbeing – such as sleep, physical health, activity, nutrition and relaxation – alongside evidence‐based treatments for mental illness. Recognising the importance of nature contact, the team and patients collaborated to enhance this aspect of care.

Initial progress was slow due to daily work pressures, organisational barriers such as policies and procedures (health and safety, risk, infection control) that have not been developed with nature in mind and COVID‐19. However, it was facilitated by regular brief meetings of a core project team, partnerships with external nature‐based organisations for skill‐sharing and the inclusion of young people in the design process.

Support from the Oxford Health NHS Foundation Trust Charity and ward art therapist enabled patients to bring nature inside through artwork. Collaboration with the gardening charity TWIGS and Wiltshire Wildlife Trust improved the biodiversity and appeal of gardens. Wild walks introduced patients to woodland and open views, providing a sense of freedom, as indicated by patient feedback as part of that improvement project.

Multidisciplinary staff, including healthcare support workers, nurses, occupational therapists, family therapists, teachers and psychiatrists, attended a 6‐day Nature Connectedness training by Natural Academy, NatureWell. Semi‐structured interviews with staff who attended this training (*n* = 8) highlighted the importance of discreet NBAs (e.g. activities that directly based on engaging with nature as opposed to being in a natural environment with indirect benefits) and their integration into existing practices to enhance psychological safety and therapeutic engagement. Key factors include involving patients in co‐designing NBAs, addressing practical considerations for organising larger activities and creating a culture that recognises the benefits for staff (Hunt et al., 2022). Staff also discussed the benefits of such training and practice, suggesting that it may have benefits for staff burnout (Hunt et al., 2022; Mitchell et al., 2024).

### Cornwall's ‘CAMHS Goes Wild’ project: Comprehensive integration of NBAs

CAMHS Goes Wild (CGW) is a pioneering initiative in Cornwall designed to integrate NBAs into clinical practice. Initial evaluations with Cornwall CAMHS staff, in collaboration with the Cornwall Wildlife Trust, indicated a desire to incorporate NBAs to maximise CYP outcomes and benefit staff wellbeing. NBAs are being integrated into practice through outdoor mindfulness sessions aimed at emotional regulation, behavioural activation activities such as beach visits and incorporating nature indoors with plants in waiting areas, nature‐themed murals in clinic rooms and natural objects to facilitate therapeutic conversations.

Recognising the need for cultural change and the limitations of the services estate model laid the foundation for directorate approval. Further exploration through Public and Patient Involvement and Engagement (PPIE) events and a mixed‐methods research study focused on staff experience identified anticipated barriers and benefits through questionnaires and interviews.

A survey of 97 respondents found that over a third reported moderate to high levels of work‐related burnout, negatively affecting job satisfaction, while about a quarter had wellbeing scores indicative of mild to severe depressive illness (Mareva et al., 2024). These findings, which highlighted the significant impact of burnout and mental health concerns on staff wellbeing, reinforced the need for interventions such as NBAs to enhance staff wellbeing and support cultural change within the service. Studies by Hunt et al. (2022) and Tambyah, Olcoń, Allan, Destry, and Astell‐Burt (2022) highlight that the integration of NBAs can be challenging but is increasingly recognised as beneficial for both staff and service users within healthcare settings, reinforcing the need for organisational support and a cultural shift. The insights gained from the study, alongside the growing evidence of the benefits of NBAs, informed implementation plans, strengthening the potential for integrating NBAs as a key part of the service's long‐term strategy (Mitchell et al., 2024).

Aligned with major and timely strategies with a focus on sustainability, CGW became an independent entity (e.g. separate governance and decision‐making structures). By showcasing results and successful training facilitated by Natural Academy, NBAs were included as a core component of care. NBAs are now part of the core mandatory training for all staff, allowing a universal approach to children regardless of need.

Securing NBAs within standard policies, mandatory training and a community of practice, CGW has ensured the sustainability of the project and foundation for further development as clinicians gain confidence. Another success is the creation of engaging patient and carer participation events. CYP were involved in PPIE activities to explore the acceptability of the research, prioritise important outcome measures and give voice to patients in discussions with decision‐makers and commissioners.

### Alder Hey's FRESH CAMHS project: Examples of nature‐based activities and social prescribing within a hospital setting

Over the past 4 years, the FRESH CAMHS initiative at Alder Hey Children's NHS Foundation has become essential for CYP. Since COVID‐19, the demand for CAMHS services has increased locally and nationally, with many young people experiencing higher levels of anxiety, low mood and mental distress. Traditional service models that are typically clinic‐led and procedural have been less accessible for some, prompting FRESH CAMHS to adopt a social prescribing model with third‐sector partners. Key programmes included:

1. Nature Well Sessions: Developed by Natural Academy, these sessions use nature‐based activities and mindfulness to support mental health. Activities include guided meditation, connecting with nature and making bird feeders. Held in the local park, these sessions are popular and have led to CAMHS staff training.
2. Forest School Sessions: Started before the pandemic, these sessions offer therapy in a natural setting, ideal for young people who prefer active engagement. Located in wooded areas of the park, they promote play, exploration and creativity. These sessions are particularly beneficial for those with neurodevelopmental conditions.
3. Walk and Talk Sessions: Introduced during the pandemic, these outdoor sessions help to facilitate better conversations and emotional regulation.
4. Growing Well: This horticulture therapy takes place in the Chelsea Garden, providing a creative space for young people to plan and cultivate garden projects, enhancing their physical and mental health.

Externally, partnerships with city organisations offer activities like fishing, pottery and sports, helping young people connect socially, enhance self‐esteem and reduce isolation. Early feedback from young people and parents has been positive. The project team is collecting data to show the importance of nature‐based therapeutic work for CYP.

## Reflections across the three projects

Integrating NBAs into practice involves coordinating service transformations and pathway reviews, aligning with the i‐Thrive model for personalised care (Wolpert et al., 2019) and integrating nature into care plans and eco‐biopsychosocial formulations. Case studies show common themes, including teamwork across disciplines, nature‐based staff training and offering a variety of NBAs. External partnerships and internal resources supported a wide range of activities. These insights inform the next section on effective NBA implementation.

Focusing on the importance of equity and accessibility to NBAs in healthcare, the case studies demonstrated how offering a variety of activities – from small gestures like indoor plants to outdoor walks and nature days – enabled CYP from all backgrounds to engage with nature in ways meaningful to them. This wide range of strategies ensured that CYP could continue exploring NBAs, regardless of the availability of natural spaces or opportunities in their home settings, promoting more equitable access to the benefits of nature.

## Operationalising an eco‐biopsychosocial approach and NBAs in CYP health services

Table 1 outlines the practicalities learned across the three case studies discussed for whole‐system considerations for integrating NBAs into practice.

**Table 1:** Factors, key recommendations and case study examples for integrating NBAs into practice

Factor	Key recommendations	Case study examples	References
Healthcare policies	Policies should underpin standard operating procedures with an eco‐biopsychosocial approach for holistic CYP care at a system level Integrate NBAs and nature‐based strategies into sustainable practices, promoting environmental stewardship Ensuring sustainability is a strategic priority	In Case Study 1, policies were developed to support nature‐based activities, ensuring alignment with health and safety standards In Case Study 2, policies were adapted to integrate nature‐based approaches into care pathways, emphasising sustainability	Hunt (2024), Tambyah et al. (2022)
Standard operating procedures (SOPs) implementation	Incorporate NBAs into routine practice Schedule regular outdoor activities (gardening, nature walks, outdoor games) with specific times for consistency Include safety guidelines, risk assessments, first aid protocols and accessibility for all CYP Evaluate NBAs across populations and seasons Transition from isolated projects to regular operations for consistency and a staff framework	In Case Study 1, SOPs were established to schedule regular outdoor activities, ensuring consistency In Case Study 2, SOPs were introduced to standardise nature‐based practices across varied settings, partnerships and resources	Hunt, Bailey, et al. (2021)
Clinician role	Model positive risk‐taking through shared risk–benefit analyses Use empirical and experiential evidence to support NBAs Compile editable risk assessment templates in a shared space for consistency and oversight Leadership support is fundamental for integrated care and advocacy for NBAs	In Case Study 1, clinicians received Nature Connectedness training, which enabled them to model positive behaviours and integrate NBAs effectively In Case Study 3, clinicians actively incorporated social prescribing into treatment plans, embedding nature‐based activities into their care practices	Hunt, Bailey, et al. (2021)
System approach	Whole‐system approach enhances staff wellbeing, prevents burnout and improves recruitment/retention Strategic communication and investment in NBAs are essential for resource allocation Positive outcomes for Case Studies 1 and 2 highlight the benefits	Case Study 1 and Case Study 2 both showed the value of embedding NBAs into long‐term service strategies Case Study 2 secured directorate approval and created external partnerships to facilitate NBA integration	Chi, Gutberg, and Berta (2020)
Training and supervision	Training should cover safety, environmental awareness, specific NBAs, eco‐biopsychosocial approaches and soft skills Multi‐tier training approach for different staff needs (building awareness, supporting nature connection, NBA enactment, train‐the‐trainer models) Continuous professional development through activities and opportunities via professional bodies like the Royal Colleges Regular check‐ins, peer support groups and mentorship programmes External supervision from bodies like Natural Academy Access to up‐to‐date resources and research Encourage a culture of feedback and continuous improvement Recognise and reward staff contributions to NBAs	Case Study 1 implemented Nature Connectedness training to enhance NBA integration in practice Case Study 2 continued implementing a multi‐tiered training approach, ensuring NBA competency across all staff levels, from frontline workers to senior leaders	Hunt et al. (2022), Mitchell et al. (2024)
Culture and motivation	Leadership must champion NBAs and demonstrate commitment Involve CYP in designing NBA offerings Incorporate NBAs into staff opportunities Celebrate successes and discuss feedback Regular engagement sessions and feedback loops for staff Recognise and reward successful NBA implementation Continuous improvement opportunities related to NBA co‐design and implementation Promote a culture of ‘this is what we do’ to shift the innovation curve towards earlier adoption Leaders should model desired behaviours and attitudes	In Case Study 2, demonstrated that CYP should be actively involved in designing NBAs to enhance ownership and motivation Case Studies 1–3 showed that offering a diverse range of NBAs enhances motivation and engagement among CYP and their families	Hunt, Dunn, Harrison, and Bailey (2021), Mareva et al. (2024), Wani and Ali (2015)
Clinical and estate strategies	Integrate NBAs into healthcare services Conduct risk assessments and care planning in natural environments Estate planning to create outdoor spaces for NBAs, either as passive/functional spaces or engaging areas for CYP and staff Co‐design NBAs with partners like wildlife trusts and community resources Recognise NBAs as essential for equitable and flexible opportunities for all CYP Promote holistic and culturally sensitive healthcare through group activities in natural settings	In Case Study 1, created partnerships with wildlife trusts to maintain and maximise the therapeutic value of outdoor spaces In Case Study 3, continued leveraging local outdoor spaces and external partnerships to sustain effective nature‐based therapy	Chi et al. (2020), Hunt et al. (2022), Robinson, Breed, Camargo, Redvers, and Breed (2024)
Evaluation and dissemination	Use appropriate PPIE strategies and ongoing evaluation Employ the Kirkpatrick training evaluation model to assess training effectiveness and integration into practice Iterative approach for matching proposed changes with relevant needs	In Case Study 1 demonstrated the use of semi‐structured staff interviews to refine NBA implementation based on feedback In Case Study 2 showed how to utilise staff wellbeing surveys to guide NBA integration and inform future evaluations	Hunt, Dunn, et al. (2021), Kirkpatrick and Kirkpatrick (2016), Hunt et al. (2022)

## Conclusion and call to action

This article illuminates the urgent need to embed NBAs within an eco‐biopsychosocial framework for CYP healthcare. Insights from the case studies and the resulting learning (see Table 1) point to key areas for future research and improvements in implementing NBAs in healthcare for CYP.

Current work identifies the need to adapt healthcare policies and SOPs to better support NBAs, ensuring both consistency and safety. The role of clinician training and collaboration with external partners is also recognised as fundamental to enhancing the delivery of these approaches. Understanding how NBAs can be embedded into healthcare culture, motivate staff and CYP, and maximise the therapeutic value of outdoor spaces is essential for future development.

Ongoing evaluation, including feedback from staff and CYP, will be key to refining these approaches and ensuring their long‐term effectiveness, changing alongside patient populations and their understanding and interests in climate change and NBAs. Communities of practice, such as ours, provide an opportunity to bringing together services from different locations to share learning on this emerging topic. Increased satisfaction and creativity can enhance wellbeing and retention, potentially mitigating NHS staff burnout as well as improving patient experience.

Supporting these initiatives with resources and recognition sustains momentum and cultivates a resilient clinical community. Active engagement from research and innovation teams is important for robust evaluation and implementation, aligning with personalised care. NBAs benefit CYP and their families, improving healthcare and recognising the importance of environmental stewardship that CYP and families must preserve natural environments and the benefits it brings. Securing large‐scale funding for equitable partnerships is essential to meet the needs of CYP but also to address the health impacts of the climate emergency and embed environmental stewardship as a core principle in healthcare services.

The time to act is now. Integrating NBAs within an eco‐biopsychosocial framework promises effective, equitable CYP healthcare, benefiting the wider system.

## Funding information

Funding was provided for the CAMHS goes Wild project by NIHR Applied Research Collaboration South West Peninsula.

## Conflict of interest statement

Hayes and Mitchell are supported by the National Institute for Health Research Applied Research Collaboration South West Peninsula. The views expressed in this publication are those of the authors and not necessarily those of the National Institute for Health Research or the Department of Health and Social Care. Mellor is employed by Natural Academy to deliver NatureWell training.

## Ethics statement

This article does not report any primary data, so ethical considerations were not necessary.

## Data Availability

Data sharing is not applicable to this article as no datasets were generated or analysed during the current study. Data that are referenced as part of the case studies are either published and cited or forms part of ongoing projects.
